# The application of a social cognition model in explaining fruit intake in Austrian, Norwegian and Spanish schoolchildren using structural equation modelling

**DOI:** 10.1186/1479-5868-4-57

**Published:** 2007-11-14

**Authors:** Camilla Sandvik, Rolf Gjestad, Johannes Brug, Mette Rasmussen, Marianne Wind, Alexandra Wolf, Carmen Pérez-Rodrigo, Ilse De Bourdeaudhuij, Oddrun Samdal, Knut-Inge Klepp

**Affiliations:** 1 Department of Nutrition, Faculty of Medicine, University of Oslo, Norway; 2 Research Centre for Health Promotion, Faculty of Psychology, University of Bergen, Norway; 3 Section of Psychiatry, Department of Clinical Medicine, Faculty of Medicine, University of Bergen, Norway; 4 EMGO Institute, VU University Medical Center, Amsterdam, The Netherlands; 5 Institute of Public Health, Department of Social Medicine, Faculty of Health Sciences, University of Copenhagen, Denmark; 6 Austrian Agency for Health and Food Safety, Vienna, Austria; 7 Community Nutrition Unit of Bilbao, Bilbao, Spain; 8 Department of Movement and Sport Sciences, Faculty of Medicine and Health Sciences, Ghent University, Ghent, Belgium

## Abstract

**Background:**

The aim of this paper was to test the goodness of fit of the Attitude – Social influence – self-Efficacy (ASE) model in explaining schoolchildren's intentions to eat fruit and their actual fruit intake in Austria, Norway and Spain; to assess how well the model could explain the observed variance in intention to eat fruit and in reported fruit intake and to investigate whether the same model would fit data from all three countries.

**Methods:**

Samples consisted of schoolchildren from three of the countries participating in the cross-sectional part of the Pro Children project. Sample size varied from 991 in Austria to 1297 in Spain. Mean age ranged from 11.3 to 11.4 years. The initial model was designed using items and constructs from the Pro Children study. Factor analysis was conducted to test the structure of the measures in the model. The Norwegian sample was used to test the latent variable structure, to make a preliminary assessment of model fit, and to modify the model to increase goodness of fit with the data. The original and modified models were then applied to the Austrian and Spanish samples. All model analyses were carried out using structural equation modelling techniques.

**Results:**

The ASE-model fitted the Norwegian and Spanish data well. For Austria, a slightly more complex model was needed. For this reason multi-sample analysis to test equality in factor structure and loadings across countries could not be used. The models explained between 51% and 69% of the variance in intention to eat fruit, and 27% to 38% of the variance in reported fruit intake.

**Conclusion:**

Structural equation modelling showed that a rather parsimonious model was useful in explaining the variation in fruit intake of 11-year-old schoolchildren in Norway and Spain. For Austria, more modifications were needed to fit the data.

## Background

Several different models and theories have been used in studies trying to predict intake of fruit and vegetables in children and adolescents. Social Cognitive Theory, Theory of Planned Behaviour, Social Learning Theory, Problem Behaviour Theory and the Stages of Change Model have all been applied (see [1] for an overview and systematic review of studies). Some of these studies included direct tests of specific theoretical models while others only used theory to guide the analytical approach. However, Rasmussen and colleagues [1] concluded that a large majority of studies carried out on potential determinants of fruit and vegetable intake lacked a clear theoretical basis.

The Pro Children study is a school-based study designed to understand and promote consumption of fruit and vegetables among schoolchildren across nine European countries. In line with state-of-the-art health promotion, the study chose a problem-driven approach. It included constructs from different behavioural theories when developing its framework and choosing which personal and environmental determinants of fruit and vegetable intake to include [2]. However, the Attitude – Social influence – self-Efficacy (ASE) model [3,4] was the main model used to inform personal and environmental determinants of fruit and vegetable intake.

The ASE-model is one of the social cognition models commonly used in predicting and explaining health behaviour. According to de Vries and colleagues [4,5], it may be considered as an extension of the Theory of Reasoned Action (TRA) [6]. It integrates the two factors of the TRA (attitudes and subjective norms) with the self-efficacy concept from Bandura's Social Learning Theory [7]. Although the model resembles the Theory of Planned Behaviour (TPB) [8], it has evolved as a separate model, with a different methodological nature [9].

Only a few studies have tested the ability of social cognition models to explain children's fruit and vegetable intake. As far as we know, no studies have yet applied the ASE-model to this issue. The ASE-model may be well suited for this purpose as it applies direct measurement of attitudes, social influence and self-efficacy [9,10]. A recent review suggests that this can result in similar or even better predictions than using belief-valuation-combinations [11]. Also, the concept of subjective norms in TPB has been replaced with a broader definition of social influences in the ASE-model [5]. This may be particularly appropriate to explain children's behaviour since a recent review suggests that children's eating behaviours are strongly influenced by their social environments [12].

**Figure 1:**
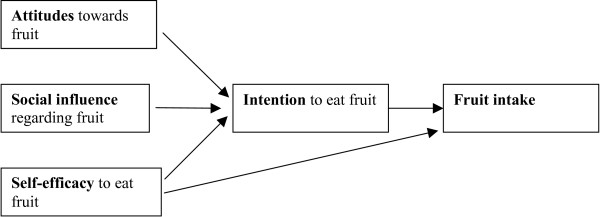
The ASE-model. A model for determining factors predictive of fruit and vegetable consumption (adapted from Lechner, 1998).

The ASE-model (see Figure 1) proposes that fruit and vegetable consumption is primarily a function of motivation or intentions, as do TRA and TPB. Three main psychosocial factors have been identified which predict intentions: attitudes, social influence and self-efficacy. A person's attitude towards fruit and vegetable consumption is a result of the expected consequences from this behaviour. Social influence is a result of subjective norms, examples from important others (modelling) and direct social support and pressure related to fruit and vegetable intake. Self-efficacy is the result of a person's subjective assessment of his or her abilities and possibilities related to fruit and vegetable intake. It is assumed that there is a direct influence from self-efficacy to behaviour [9,13]. This has been confirmed in studies on smoking in adolescents [4,5,8] and on fruit and vegetable intake in adults [13].

The relationships between attitudes, social influence and self-efficacy have not been specified in the paper which presents the ASE-model [3], but de Vries and colleagues [4,5] allowed attitude, subjective norm and self-efficacy to correlate freely when applying the model to smoking data. Kok and colleagues stated that the three constructs are not completely independent of each other [14], and the components are typically positively correlated. The relation between the three factors and intention is assumed to be linear. At least two studies [4,8] have tested for interaction effects between attitudes, social influence and self-efficacy, but did not find a significant effect, either on intention or on behaviour. External variables, such as socio-demographic factors, are expected to influence behaviour through behavioural determinants and intention [13].

The studies that have tested the predictive value of social cognition models in explaining children's fruit and/or vegetable intake, have often used varying multiple regression techniques [1]. Some researchers have also applied structural equation modelling techniques (SEM) [15-19]. However, only one of these studies explicitly tests one of the social cognition models, namely TPB [17].

In this paper, we want to explore the fit and the strength of the ASE-model in explaining fruit intake in schoolchildren in three European countries. In particular, we want to test the goodness of fit of the ASE-model in explaining schoolchildren's intentions to eat fruit and their actual fruit intake in Austria, Norway and Spain. We want to assess the unique contribution each variable makes to intention to eat fruit and to subsequent fruit intake. We also want to assess how well the predictors in the model can explain the observed variance in intention to eat fruit and in reported fruit intake. In addition, we want to see whether the same model fit data from all three countries. We use structural equation modelling as this is considered to be the best methodological and theoretical approach for this type of study [20].

As previously reported [21], fruit intake differed substantially between schoolchildren in the nine European countries participating in the Pro Children study. The number of children responding positively to various explanatory factors regarding fruit and vegetable intake also differed markedly between countries [22]. We therefore decided to test the model on samples from three of the countries in the Pro Children study, a northern country (Norway), a central-European country (Austria), and a Mediterranean country (Spain), to see whether the same model would fit data from all three countries.

## Methods

### Design

Samples of schoolchildren from three countries (Austria, Norway, and Spain) participating in the cross-sectional part of the Pro Children project were used in this study. The target group was 11-year-old schoolchildren, and school classes formed the sampling unit (fifth or sixth grade). Only children born in 1991 and 1992 were included in this study. The Norwegian (N= 1142; response rate 89.5%) and the Spanish samples (N = 1297; response rate 94.7%) were nationally representative while the Austrian sample (N = 991; response rate 95.3%) was representative for the Eastern region of the country, which covers 42% of the full population. Mean age was 11.3 to 11.4 years. Table 1 provides further information on the samples. Self-administered questionnaires were completed during one school lesson. Data were collected during October-December 2003. Ethical approval for the Pro Children study was obtained from the relevant ethics committees in the three countries. Responses were treated anonymously and respondents were told that their responses were confidential. Written informed consent was obtained from parents of the children in Austria, whereas the Norwegian and Spanish study used passive, informed parental consent. Further details on the data collection procedure as well as on the subjects participating in the cross-sectional study have been published elsewhere [21].

**Table 1:** Characteristics of the samples

		**Country**	
	**Austria**	**Norway**	**Spain**
**Sample size**	991	1142	1297
**No (%) of boys**	500 (50.5)	568 (49.7)	694 (53.5)
**Mean age (SD)**	11.4 (.41)	11.3 (.29)	11.4 (.42)
**Age range**	10.8–12.8	10.8–12.3	10.8–12.8
Number of classes/schools*	73 classes/23 schools	73 classes/52 schools	64 classes/37 schools
Response rate*	95.3%	89.5%	94.7%

*Note: Number of classes/schools, and response rate is based on the original Pro Children samples. For this study, children born in 1990, 1993 and 1994 were excluded from the samples.

### Measures and constructs

The questionnaire included the following items assessing key ASE-constructs of Attitudes, Social influence, Self-efficacy, Intention and Behaviour (fruit intake). Attitudes were measured with two items: *To eat fruit every day gives me more energy*, and *to eat fruit every day makes me feel good*. A five-point Likert scale was applied, going from 'I fully agree' to 'I fully disagree' for both items. As Liking and Preferences can be considered to be dimensions of a more inclusive attitude construct, sub-constructs measuring these concepts were included in the initial factor analysis to see if they contributed to a meaningful attitude construct together with the original attitude items. Liking was assessed by the items *I like to eat fruit every day*, and *fruit tastes good*, and Preferences were measured by the question: *Which of the following fruits do you like or dislike*, followed by a list of 12 fruits and using response categories from 'I like very much' to 'I dislike very much'.

Social influence was assessed by three items measuring 'Descriptive norms' or 'Modelling': *My mother/father/best friends eat fruit every day *(same Likert scale as for the attitudinal items); two items that measured 'Active parental encouragement': *My mother/father encourages me to eat fruit every day*, with response categories as before. In addition, the 'Demand family rule' was measured by a single item: *Do your parents demand that you eat fruit every day*, whereas the 'Allow family rule' was assessed by: *Are you allowed to eat as much fruit as you like at home? *'Parental facilitation' was measured by the item: *Does your mother or father usually cut up fruit for you in between meals? *The last three items all had five response categories from 'yes, always' to 'never'.

Two items were designed to measure Self-efficacy: *It is difficult for me to eat fruit every day*, and *if I decide to eat fruit every day, I can do it*. Intention was measured by one item: *I want to eat fruit every day*. Again, response categories ranged from 'I fully agree' to 'I fully disagree'.

Fruit intake was measured with the fruit item from a validated fruit and vegetable food frequency questionnaire: *How often do you usually eat fresh fruit? *Eight response categories ranged from 'never' to 'every day more than twice a day'. The development and pilot-testing of the determinant part as well as the fruit intake part of the questionnaire used in the cross-sectional study of the Pro Children project have been described in detail elsewhere [23,24].

### Analyses

**Figure 2:**
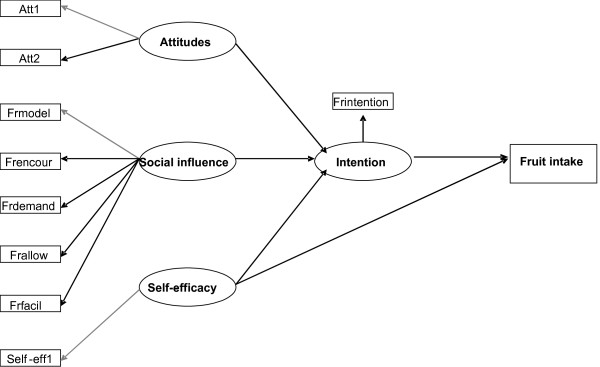
Structure of the initial model.

The constructs and variables included in the cross-sectional study were scrutinised to re-assess which items belonged where in the ASE-model, and to specify the initial model (see Figure 2). Descriptive analyses were carried out to test for non-normality in data. As suggested by Kline [25], we chose to apply cut-off values of 3.0 for skewness and 8.0 for kurtosis. We also ran bivariate correlation analyses between the independent variables to test for multicollinearity. The structure of the latent variables included in the model was then tested by exploratory factor analysis. This was carried out in SPSS, using principal axis factoring as the extraction method and varimax rotation. For self-efficacy, we ran inter-item correlations, since the construct consisted of two items only.

PRELIS was used to prepare data for the structural equation models. First, missing values were imputed using Imputation by Matching (IM). This method is suitable when imputing ordinal variables such as Likert scale data. Cases with complete data that match or which have similar patterns of scores on intact variables to cases with missing values, are used to impute values on variables with missing data. In contrast to the Expectation Maximization (EM) method of imputation, which does not keep variables as ordinals, this method gives equal values as already specified for the categories. Cases with missing data after IM were deleted listwise. Polychoric correlations and asymptotic covariance matrices were then estimated for all samples with Robust Maximum Likelihood estimation. This procedure transforms the observed ordinal variables into latent underlying continuous variables that are normally distributed, with metric and variance. The thresholds were set equally by computing for the total sample. For single item measures, pre-specified residual variances were added, to take measurement error in these variables into account. For this procedure, an alpha-level of .80 was chosen, as this would represent a satisfactory level for a scale with sufficient number of variables and magnitude of covariances. Structural equation modelling techniques were used with LISREL software, version 8.8 [26].

The Norwegian sample was used to test the structure of the observed and latent variables in the model, to make a preliminary assessment of the model fit, and to modify the model to increase goodness of fit with the data. Both the initial and modified models were then applied to the Austrian and Spanish samples to assess model fit. Non-significant relations were removed and the model was re-estimated for each country until the models achieved close fit and all relations were statistically significant. After the final model was fitted to data, gender- and age specific analyses were carried out to see whether the structure model(s) was confirmed for boys and girls, and for children born in 1991 and 1992.

*Measures of model fit: *Since indexes addressing goodness of fit reflect different aspects of model fit, Kline recommends reporting several fit indicators [25]. The χ^2 ^has traditionally been used to test the hypothesis that the relationships suggested in the model provide a plausible explanation of the data, i.e. how well the proposed model structure fits the structure in the observed data set(s). Ideally, it should be non-significant. However, most models are slightly mis-specified, i.e. they only fit the data approximately [27]. So, when sample sizes are large, measurement errors tend to result in significant chi-squares [18,27]. However, differences in chi-square are used to evaluate differences between nested models in this study, as in the case of deleting or adding a parameter. Satorra-Bentler scaled χ^2 ^was used to adjust for ordinal data. This measure is also robust in relation to non-normality in data. RMSEA, with 90% confidence intervals, and the probability of achieving close fit (RMSEA < .05) is also reported. This is the likelihood of getting an RMSEA below .05 when repeating the model fit procedure on an indefinite number of samples. Other fit measures presented are GFI, AGFI, NNFI, and Standardized Root Mean Square Residual (RMR). Both AGFI and RMSEA are parsimonious fit measures.

The RMSEA is relatively insensitive to sample size, since it is a population-based index. It has an explicit parsimony adjustment [28], as it simulates the error per degree of freedom in the model. There have been discussions of how low RMSEA should be to define a good fit. Browne and Cudeck [29] suggest RMSEA <.05 as close fit and values beyond .10 as poor.

## Results

### Descriptive statistics and inter-correlations

Table 2 gives an overview of the distribution of fruit intake in the total sample and in the different countries. An overview of the mean scores, standard deviations, number of items and range for the constructs in the ASE-model is presented in Table 3. Screening for skewness and kurtosis showed that one of the self-efficacy items and the item measuring the 'Allow family rule' had values above the chosen cut-offs for the Austrian sample. Further, the self-efficacy item was moderately skewed for the Norwegian and Spanish sample. The estimation method chosen and the use of Satorra-Bentler scaled χ^2 ^takes non-normality in data into account. Table 4 and 5 provide bivariate correlations between all constructs for Norway, Austria and Spain respectively, and show that none of the inter-correlations between the independent variables were above .60. Most variables were positively and significantly correlated to fruit intake, except three of the social influence sub-constructs for the Austrian sample.

**Table 2:** Fruit intake distribution for the total sample and for the separate countries (based on valid percent)

	**Total sample**	**Austria**	**Norway**	**Spain**
**Never**	2.0	1.2	1.1	3.3
**Less than one day/week**	4.9	3.9	7.3	3.7
**One day/week**	8.2	5.0	11.3	7.8
**2–4 days/week**	28.8	25.0	32.6	28.3
**5–6 days/week**	15.0	15.5	17.4	12.4
**Every day, once**	19.2	20.9	14.8	21.7
**Every day, twice**	11.5	14.1	7.9	12.8
**Every day, more than twice**	10.5	14.5	7.6	9.9

**Table 3:** Number of items, range, N, means (SD) for the constructs in the ASE-model

			**Austrian sample**			**Norwegian sample**			**Spanish sample**		
Construct/Scale	No of items	Range	N	Mean	(SD)	N	Mean	(SD)	N	Mean	(SD)
**Attitudes**											
Attitudes	2	-2/2	990	1.40	.81	1128	1.25	.80	1281	1.48	.73
**Social influence**											
Modelling	3	-2/2	981	1.00	.75	1130	.56	.82	1278	.98	.77
Active parental encouragement	2	-2/2	977	.55	1.29	1134	.20	1.28	1269	1.05	1.12
Demand family rule	1	-2/2	956	-.15	1.28	1120	-.65	1.13	1241	.72	1.23
Allow family rule	1	-2/2	959	1.82	.56	1126	1.44	.83	1234	1.36	1.09
Parental facilitation	1	-2/2	974	.24	1.24	1106	-.59	1.03	1261	-.19	1.33
**Self-efficacy**											
If I decide to eat fruit every day, I can do it	1	-2/2	972	1.73	.75	1127	1.63	.84	1273	1.51	.89
**Intention**											
I want to eat fruit every day	1	-2/2	974	1.18	1.05	1128	1.31	.99	1272	1.07	1.10
**Fruit intake**											
Fruit intake	1	0/7	987	4.41	1.70	1129	3.72	1.64	1288	4.08	1.76

Note: All differences in means are statistically significant at a .01 level, except for Attitudes between Austria and Spain; Modelling between Austria and Spain; Allow family rule between Norway and Spain; Self-efficacy between Austria and Norway, and Intention between Austria and Norway and between Austria and Spain.

**Table 4:** Pearson's correlation between all scales for the Norwegian sample

	Attitudes	Model	Encourage	Demand	Allow	Facilitate	Self-efficacy	Intention	Intake
Attitudes	1								
Modelling	.24**	1							
Active parental encouragement	.27**	.45**	1						
Demand family rule	.18**	.31**	.48**	1					
Allow family rule	.10**	.18**	.18**	.12**	1				
Parental facilitation	.19**	.28**	.34**	.35**	.13**	1			
Self-efficacy+	.15**	.13**	.13**	.07*	.14**	.02	1		
Intention	.44**	.29**	.30**	.19**	.13**	.19**	.22**	1	
Fruit intake	.27**	.36**	.26**	.26**	.12**	.18**	.12**	.40**	1

* Correlation significant at the 0.05 level

** Correlation significant at the 0.01 level.

+ It is the correlation for the single-item measure of self-efficacy that is reported here.

**Table 5:** Pearson's correlation between all scales for the Austrian and Spanish samples (Austrian data below the diagonal line, Spanish above)

	Attitudes	Model	Encourage	Demand	Allow	Facilitate	Self-efficacy	Intention	Intake
Attitudes		.28**	.23**	.15**	.14**	.15**	.36**	.49**	.24**
Modelling	.28**		.32**	.23**	.05	.13**	.22**	.33**	.23**
Active parental encouragement	.08**	.25**		.49**	.17**	.24**	.14**	.25**	.10**
Demand family rule	.01	.13**	.46**		.14**	.25**	.06*	.12**	.11**
Allow family rule	.09**	.04	.08*	-.02		.11**	.12**	.15**	.06*
Parental facilitation	.14**	.28**	.23**	.21**	.05		.09**	.19**	.20**
Self-efficacy+	.27**	.16**	.06	.01	.16**	.12**		.47**	.25**
Intention	.48**	.30**	.05	-.01	.09**	.15**	.32**		.42**
Fruit intake	.29**	.24**	-.03	-.00	.04	.22**	.19**	.48**	

* Correlation significant at the 0.05 level

** Correlation significant at the 0.01 level.

+ It is the correlation for the single-item measure of self-efficacy that is reported here.

### Exploratory factor analyses of latent constructs

Exploratory factor analysis for the attitude concept including the two original attitude items, the two 'liking' items and the 'preferences' items did not result in a meaningful factor structure as more than 25 iterations were required. Therefore extraction was terminated. In the subsequent SEM analyses, we only included the two original attitude items as a measure of the attitude construct. Inter-item correlation for these was .57 for the Norwegian sample.

Exploratory factor analysis for 'social influence' for the Norwegian sample resulted in one factor with an eigenvalue above 1, explaining 44% of the total variance. All sub-constructs had factor loadings above .50, except for the 'allow family rule'. In the subsequent SEM analyses, social influence was therefore included as a latent variable, defined by modelling, active parental encouragement, demand family rule, allow family rule, and parental facilitation.

For self-efficacy the inter-item correlation was low (.17). We chose therefore not to use the item *It is difficult for me to eat fruit every day *in further analyses. This item was reverse-coded, and we suspect this caused the low correlation [30]. Hence, self-efficacy was measured with one item only.

### Structural equation modelling

**Figure 3:**
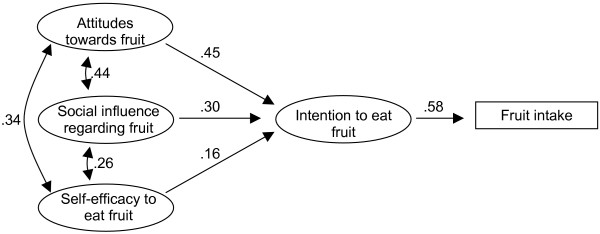
The final model fitted to the Norwegian sample. To simplify the presentation, the factor loadings and residual values are omitted.

Applying the original ASE-model to the **Norwegian data **resulted in a very good model fit for the full model. However, the relation from self-efficacy to intake was not empirically supported in the Norwegian sample (-.04 p > .05), so we deleted it from the model. The goodness of fit statistics for the re-estimated model was: χ^2 ^= 69.82, (df = 31, p < .001), GFI = .97, AGFI = .95, NNFI = .99, standardized RMR = .04, RMSEA = .03. 90% CI for RMSEA was .02–.04, p-value for close fit (RMSEA_<.05_) was 1.00. The final model is presented in Figure 3. This model gave an explained variance of 51% for intention to eat fruit every day, and 34% for reported fruit intake. Gender specific analyses without constrained parameters for factor loadings and parameter estimates between the predictors showed one difference in structure models for boys and girls in Norway: For boys, the relation between self-efficacy and intention was non-significant (.08). There were also minor differences in the magnitude of the parameters. Neither model gave empirical support for a direct relation between self-efficacy and fruit intake. Age specific analyses could not be carried out for the Norwegian sample as the 1991 group consisted of 12 children only.

**Figure 4:**
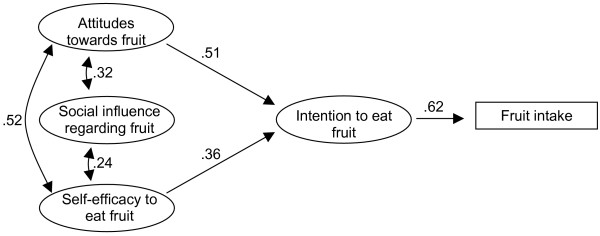
The final model fitted to the Austrian sample. To simplify the presentation, the factor loadings and residual values are omitted.

When we applied the original ASE-model to the **Austrian **data, model fit was poor. The coefficient for the relation between self-efficacy and fruit intake was -.10 (non-significant) and the relation between social influence and intention was .02 (non-significant). The modification index (MI) indicated that several residual covariances between the social influence indicators had to be freed for estimation (frmodel – frdemand, frdemand – frallow, frallow – fruitfac, and frmodel – frallow; cf. Figure 2). We applied a revised model with these relations and without the two structure relations. MI suggested cross-factors between the exogenous latent variables, but these were not freed since this was contrary to the theoretical specification. The re-estimated model achieved a better fit: χ^2 ^= 99.13, (df = 28, p < .001), GFI = .94, AGFI = .89, NNFI = .96, standardized RMR = .08, RMSEA = .05, 90% CI for RMSEA = .04–.06, p-value for close fit (RMSEA_<.05_) = 0.33. So, the model that fitted the Austrian data best did not have a significant relation between social influence and intention. Further, there was no empirical support for a direct relation between self-efficacy and intake in the Austrian sample. Figure 4 shows the relation from each of the exogenous variables to intention, the relation between intention and intake, and gives an overall graphic presentation of the final model for the Austrian data. This model could explain 59% of the variance in intention to eat fruit, and 38% of the variation in fruit intake. For Austria, we also ran gender specific analyses comparing a constrained multi-sample model with a model where the parameters were allowed to vary freely. The constrained model fared no worse than the other, so there is no empirical support for differences in model structure between the genders in the Austrian sample. The age specific analyses showed that adapting a similar model did not fare significantly worse than adapting separate models for children born in 1991 and 1992 for the Austrian sample.

**Figure 5:**
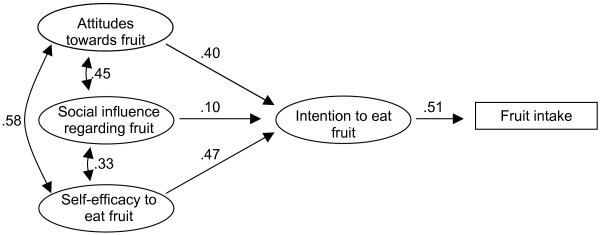
The final model fitted to the Spanish sample. To simplify the presentation, the factor loadings and residual values are omitted.

When the original ASE-model was applied to the **Spanish data**, model fit was good. However, as for the Norwegian data, the relation from self-efficacy to intake was not statistically significant (-.05, p >.05). The goodness of fit statistics for the re-estimated model was: χ^2 ^= 118.04 (df = 31, p < .001), GFI = .96, AGFI = .94, NNFI = .97, standardized RMR = .06, RMSEA = .05, 90% CI for RMSEA = .04–.06, p-value for close fit (RMSEA_<.05_) = 0.57. The model that fitted the Spanish data best is presented in Figure 5. The final model could explain 69% of the variance in intention to eat fruit for the Spanish children, and 27% of the variation in fruit intake. Gender specific analyses did not reveal any significant difference in model structure for boys and girls in Spain. A multi-sample analysis where the paths were constrained did not fare significantly worse than when the paths were allowed to vary freely. So, there was support for equal paths and equal relations between genders for the Spanish sample. Further, age specific analyses for Spain showed that adapting a similar model for children born in 1991 and 1992 did not fare significantly worse than adapting separate models.

We did not find support for applying an identical model on data for all samples and could not carry out multi-sample SEM analyses to investigate whether the relations between the different constructs were identical or not between countries. Also, the pattern of which construct was the strongest correlate of intention to eat fruit every day, varied between countries. Attitude was strongest in Norway and Austria, but for Spain, self-efficacy was strongest. However, the effect of attitude was strong for all three countries. Social influence appeared to be of medium strength in Norway, weak in Spain and non-existent in Austria. Self-efficacy was the strongest correlate in Spain, of medium strength in Austria, and the weakest correlate in Norway.

## Discussion

Applying the ASE-model to the data from the cross-sectional survey of the Pro Children study showed that the model fitted the data well for two of three countries, but without the direct relation from self-efficacy to behaviour. The results showed that the model could explain between 51 and 69% of the variance in intention to eat fruit, and 27 to 38% of the variance in reported fruit intake in children. In comparison, other studies have applied different methods and analysis strategies, and achieved between 26 to 31% explained variance in intention to eat (more) fruit [17,31] and between 7 and 34% for fruit (and vegetable) intake in adolescents [17,31-33]. Furthermore, Brug and colleagues found that 47% of the variance in intention to eat fruit and 21% of the variance in fruit intake could be accounted for in adults [13]. This is in line with what Baranowski and colleagues [34] concluded in an earlier review; that studies on psychosocial correlates to fruit and vegetable intake tended to explain less than 30% of the variance in intake.

These findings, despite the fact that some potential correlates were assessed with single items, support the assertion that the ASE-model is well suited to explore correlates and potential determinants of eating behaviour (fruit intake specifically) in 11- to 12-year-old schoolchildren. It seems that we were able to explain more of the variance in intention than previous studies, and slightly more of the variance in intake. Choice of analytical approach may have contributed to this as structural equation modelling accounts for measurement error to a larger extent than multiple regression [20], and may produce higher estimates of explained variance. SEM also provides the possibility of adapting the model to each individual dataset. This may increase the level of explained variance somewhat. Finally, by correcting for measurement error in single items and running SEM-analysis adapted to ordinal data, a slightly higher R^2 ^may be achieved. However, by running the same analysis on our samples while not correcting for measurement error and not taking ordinal level data into account, we could still explain between 30 and 44% of the variance in the intention to eat fruit, and 20 to 25% of the variance in fruit intake.

Furthermore, some of the previous studies may have underestimated the strength of relation between intention and intake leading to a lower R^2^, for instance by measuring intention to eat *more *fruit and vegetables [17] or measuring intention to change behaviour [31], and relating this to regular intake. Violating the principle of compatibility [35] may lead to lower predictive power [36]. In this study, we measured all correlates and intake in relation to consumption 'every day', aiming at a better correspondence between correlates and intake. Also, some of the previous studies were predicting fruit and vegetable intake as one behaviour [17,33]. Since fruit and vegetable consumption can be seen as different behaviours, influenced by different factors [37], this could have reduced the predictive power of these studies.

However, the ASE-presumed direct path from self-efficacy to intake behaviour was not confirmed in our study. This may be due to the low variation in answers to the self-efficacy questionnaire item as almost all children agreed somewhat or fully that they could eat fruit every day if they decided to. Further, as self-efficacy was assessed with one item only, this result should be interpreted with caution.

### Attitudes

Attitude was the strongest correlate of intention to eat fruit every day for two out of three countries participating in this study which is consistent with previous research among adolescents [31]. Further, as in Brug et al. [13] and Lien et al. [17], but contrary to Martens et al. [31], there were no significant direct relations between attitudes and behaviour in any of the countries. The inter-item correlation between the attitude items was acceptable, and the construct fitted nicely into the overall model for all three countries.

Taste preferences have been shown to be one of the strongest and most robust correlates of fruit and vegetable intake in children and adolescents [1,18,32,33,38], and should ideally have been included in the attitude construct aiming at predicting or explaining fruit intake in children. Due to methodological constraints this was not possible in this study.

### Social influence

Social influence was the second strongest correlate to intention of eating fruit in Norway, but weak for Spain and non-significant for Austria. Furthermore, the one-factorial structure of social influence was not sufficient to explain the observed relations in the data for the Austrian sample as we had to free four of the relations between the residuals in the social influence latent construct. Also, the factor loading for parents' allowing their children to eat fruit on the social influence construct was rather low for this sample (.26). It seems that social influence was not a uni-dimensional concept for these children. Furthermore, the bivariate correlations between active parental encouragement, the demand and allow family rules, and fruit intake were non-significant and close to zero for the Austrian sample (Table 5).

One way to interpret this finding could be that Austrian children did not perceive the social influence from their parents as consistent. However, the social influence concept was set out to measure both subjective beliefs about social norms and behaviours, observed behaviours of others (or modelling), as well as direct social pressures and support [3], so it may not be reasonable to assume that the underlying construct is one-dimensional. De Vries and colleagues [5] also questioned the uni-dimensionality of the social influence concept both in the Theory of Planned Behaviour and in the ASE-model, and proposed a three-dimensional concept, consisting of social norms, perceived behaviour of others (modelling) and direct pressure. However, since we did not predefine or measure social influence as a three-dimensional concept, we could not analyse it as such in this paper. Future studies should investigate the properties and the possible dimensions of this concept further, especially since a recent review confirms that children's eating behaviours are strongly influenced by their social environments [12].

### Self-efficacy

Self-efficacy was the poorest ASE correlate of intention to eat fruit in Norway and Austria, but the strongest in Spain. For none of the countries did we find evidence for a direct influence from self-efficacy to behaviour as assumed by the ASE-model. Self-efficacy was originally measured by two items in the questionnaire, measuring domain-specific self-efficacy. However, the inter-item correlation was low in our sample, as well as in an earlier study of the reliability of the measured constructs [23]. We chose therefore not to use the item *It is difficult for me to eat fruit every day*, as it was reverse-coded and we suspected this to may have caused problems for the children. Hence, self-efficacy was measured by one item only. Although this is not an optimal strategy [39], it was considered a more appropriate methodological choice than including an unclear concept. This may, however, have affected the ability of the concept to influence behaviour both indirectly and directly.

Self-efficacy in children age 11 may not be well developed. As 11-year-old children are on the verge of puberty, cognitively they are less able to plan ahead, to focus their attention, and to think in a strategic manner than after puberty [40]. There may be large discrepancies between perceived self-efficacy and actual control for this age-group. In addition, children may have limited autonomy and influence over food choices and may be more dependent on environmental factors such as fruit availability and accessibility [2] in order to act on their self-efficacy-intention contingencies.

Further, de Vries and colleagues [4] stated that the correspondence between perceived and actual behavioural control may be less clear when behaviour is complex and dependent on several variables. Fruit consumption can be viewed as a complex behaviour, with different fruit being eaten at different times during the day and for different reasons.

As stated earlier [22], our measure of self-efficacy has deficits, and one should be cautious in interpreting the findings regarding this concept. However, findings from previous studies indicate that the effect of self-efficacy on fruit intake is largely mediated through intention [13,31,41]. Future research in this area should improve self-efficacy measures in children. This would allow us to assess whether self-efficacy is working only through intention or also has a separate, direct effect on behaviour of practical significance. Self-efficacy measures applied in future research could also be more specific, for example, asking about efficacy in skills or efficacy in overcoming barriers, and more related to specific behaviours in specific situations, as outlined by Bandura [7] and carried out by the Baranowski group. This could help children reply more accurately to questions about self-efficacy. However, the self-efficacy measure developed by the Baranowski group, a 34-item situation specific self-efficacy questionnaire for fourth- and fifth-graders in the US (see [42] for a description), had very low criterion validity against consumption (ranging from .00 to .17), and self-efficacy was a non-significant predictor of fruit and total fruit and vegetable consumption [42]. Hence, further research in this area is warranted.

### Intention to eat fruit

As we decided to use 'I want' as our intention item instead of 'I intend..' to accommodate wording to our target group, the item may have ended up closer to Bagozzi's desire concept [43] than the original intention concept. However, measures of desire, intention and expectation commonly have a very high level of correlation [44], and studies on TPB have typically employed mixed measures of intention [45]. If anything, desire tends to have a poorer prediction of behaviour than intention measures [45]. Thus, the true predictive/explanatory power of intention in these samples may be underestimated in this study.

### Differences in model structure between countries

We could not carry out multi-sample/multigroup SEM analyses to see whether the relations between the different constructs were identical in the three countries because we had to free more relations and apply a more complex model to the Austrian sample. Also, the relation between social influence and intention was not significant for the Austrian sample. This suggested that the factors affecting fruit intake among schoolchildren in the three countries differed to a certain degree. Although there are limitations in the study related to measurement in particular, this is a significant finding. It supports the importance of conducting research on children from different cultures to better determine which factors predict fruit intake in children from each of these cultures.

The different patterns of relations between the psychosocial factors and intention/behaviour were in accordance with Fishbein's assumption that the relative importance of these three psychosocial variables (i.e. attitudes, social influence or social norms, and self-efficacy) as determinants of intention will depend upon both the behaviour and the population being considered [46]. It seems that the fruit intake of Norwegian children was more influenced by what their parents said or did, whereas for Spanish children, self-efficacy was relatively more important. Perhaps this reflects different degrees of fruit availability in these countries. Sandvik and colleagues have previously shown that perceived home availability of fruit is lower among Norwegian schoolchildren than among Spanish [22]. If fruit is freely available at home, eating fruit may be more a matter of the child's own decision. Further research into availability differences and availability-self efficacy interactions is warranted.

#### Model structure and age

The three samples differed slightly in their mean age, as well as in age range. We therefore compared the models for children born in 1991 and 1992 for Austria and Spain, to explore whether some of the differences in model structure between countries could be attributed to differences in age composition across the samples. (The Norwegian 1991 sub-sample consisted of 12 children only, so these analyses could not be performed for Norway). These analyses showed that adapting a similar model did not fare significantly worse than adapting separate models regarding parameter values to children born in 1991 and 1992. So, data supported use of the same model for the youngest and oldest children in our sample. This does not rule out the idea that there may be effects of age in the samples, but they do not affect model structure to a significant degree in Spain and Austria.

#### Model structure and gender

When comparing gender specific models where the paths were constrained to models where the paths were un-constrained, we found significant gender differences in model structure for the Norwegian sample only. Lien and colleagues [17] have previously documented gender differences in model structure when applying the TPB to fruit and vegetable intake in US adolescents. Previous research has also reported gender differences in level of intake of fruit and vegetables [1,17,21], in correlates to intake [22], as well as in mediating factors [47]. The fact that we observe some differences in model structure in one of the three countries only is an important finding, which deserves further exploration. Several issues could explain this, for instance cultural differences in how gender is staged, both in the parent generation and among the children themselves. However, this is beyond the scope of this paper.

### Strengths and limitations of this study

The novel aspect and strength of this study is that structural equation modelling techniques were applied to assess model fit for the ASE-model, for assessing the unique contribution from each variable to intention to eat fruit and then to actual fruit intake, and to estimate explained variance. SEM techniques are based on adequate or large sample sizes [25], and the Pro Children study provided adequate sample sizes. In addition, as SEM provides for simultaneous testing of several variables at once and the possibility to free relations when the factor structure is unclear, the method is far better suited to test social cognition models than traditional regression. The samples used in this study were either country- or region representative samples instead of convenience samples, which have often been used in this kind of research [1]. It is likely that the results found here are valid for children in this age group for the country as a whole. However, differences related to ethnicity and different socio-economic groups have not been accounted for. Also, there were some structure differences in the models related to gender for Norway, which should be further explored. These issues need to be taken into consideration when generalising results.

Other limitations in this study are related to measurement of the potential correlates. Although internal consistencies for the multi-item measures were all above .50, some of them were not very high. Other constructs were measured with one item only. This may have affected the level of explained variance in the model, as well as other parameter estimates. Future research should aim at achieving a higher reliability by including a sufficient number of items for each construct measured. It was decided to measure each construct with only a limited number of items (often as few as one or two), since the questionnaire had to be administered within one school hour and had to cover fruit as well as vegetable intakes. Fruit intake was also measured with one item only. However, the validation study which was carried out in four of the Pro Children countries, showed a fairly good ranking of the children according to their fruit intake, based on the food frequency part [24]. In the latter study, usual fruit intake was also measured with one item, and validity of fruit juice intake was assessed separately.

In addition to the time limit, we also had to take into account the limitations in cognitive abilities of 11-year-olds, their concentration span and reading abilities. The questionnaire was therefore thoroughly tested during the development phase, and reliability was moderate to high for all constructs (range .52 to .89) with the exception of the general self-efficacy scale [23]. We also used group discussions with children at this stage, to assure that wordings and word choice were understandable and adequate for children in this age group [23].

Eliminating reverse-coded items in the analyses could be problematic, as this leaves open the possibility that the results were driven by a positive response bias across items. However, this study was carried out anonymously, and reported intake of fruit and vegetables was low [21], which may be evidence against the interplay of such an effect. Also, for some items or constructs in the questionnaire, few children had positive replies [22]. This also speaks against the existence of a general pleasing effect or positive response bias.

Although the questionnaire was developed with the aim of being fully comparative between countries (see [23] for a more detailed description), we cannot rule out the possibility of cultural differences in how items and questions were interpreted, and differences between countries in children's ability to self-report accurately based on skills, prior experience and interpretation. However, test-retest reliability scores were quite similar across the five participating countries in the test-retest sample (Norway, Spain, Denmark, Portugal, Belgium), indicating that this was not a major problem in this study [23].

As for many other studies in this field, we used a cross-sectional design. This does not allow us to draw any conclusions about causality and the direction of effects. Ideally, the ASE-model should have been tested using prospective data to assess its fit in an optimal way [34]. However, the questionnaire used in this study was both theory- and evidence-based, and validity and reliability was thoroughly tested during questionnaire development. This makes it easier to stand on solid ground when drawing conclusions from the study.

## Conclusions

Structural equation modelling showed that the ASE-model was useful in explaining fruit intake in 11-year-old schoolchildren in Norway and Spain. The explained variance was similar to or better than what has been found in other studies, even though a rather parsimonious model was applied.

However, the latent construct 'social influence' created problems, in particular in Austria, and the uni-dimensionality of this construct is questioned. Future research should look at the properties and the possible dimensions of this concept. We did not find support for the assumed direct link between self-efficacy and behaviour in the ASE-model. Further studies could investigate whether this is related to the cognitive-developmental status of children this age, or whether improved instruments could capture self-efficacy in 11-year-old schoolchildren in a better way.

Our study indicates that slightly different models were needed to fit data from different countries within Europe, suggesting that determinant patterns may be different across Europe. Although building on the same theory, interventions to increase fruit intake should be specifically tailored to the culture in which they are to be implemented, in order to achieve maximum effect.

## Competing interests

None of the authors have any affiliation, financial agreement, or other involvement with any company related to the submitted manuscript.

## Authors' contributions

CS came up with the idea together with KIK, ran descriptive and bivariate analyses, carried out exploratory factor analyses and drafted the manuscript. RG carried out the LISREL analyses for this paper and drafted the method section and part of the results. JB aided in further developing the ASE-model, offering insight on model development and references thereto. MR provided points and references for the introduction and the discussion parts of the paper. AW and CPR made substantial contribution to acquisition of data. JB, RG, MR, MW, AW, CPR, IDB, OS and KIK read and commented on several versions of the paper. CS and RG re-ran all analyses for revision, and re-drafted the paper. All authors are responsible for the reported research. All authors have participated in drafting or revising of the manuscript, and all authors read and approved the manuscript as submitted.

## References

[B1] Rasmussen M, Krolner R, Klepp KI, Lytle L, Brug J, Bere E, Due P (2006). Determinants of fruit and vegetable consumption among children and adolescents: a review of the literature. Part I: quantitative studies. International Journal of Behavioral Nutrition and Physical Activity.

[B2] Klepp KI, Pérez-Rodrigo C, De Bourdeaudhuij I, Due PP, Elmadfa I, Haraldsdóttir J, König J, Sjöström M, Thórsóttir I, Vaz de Almeida MD, Yngve A, Brug J (2005). Promoting Fruit and Vegetable Consumption among European Schoolchildren: Rationale, Conceptualization and Design of the Pro Children Project. Annals of Nutrition and Metabolism.

[B3] Kok G, Herman S, de Vries H, Parcel G, Paulussen T (1996). Social psychology and health education. European Review of Social Psychology.

[B4] de Vries H, Dijkstra M, Kuhlman P (1988). Self-efficacy: the third factor besides attitude and subjective norm as a predictor of behavioural intentions.. Health Education Research.

[B5] de Vries H, Backbier E, Kok G, Dijkstra M (1995). The impact of social influences in the context of attitude, self-efficacy, intention, and previous behavior as predictors of smoking onset.. Journal of Applied Social Psychology.

[B6] Ajzen I, Fishbein M (1980). Understanding attitudes and predicting social behavior.

[B7] Bandura A (1986). Social foundations of thought and action: a social cognitive theory. Prentice-Hall series in social learning theory.

[B8] Ajzen I, Madden TJ (1986). Prediction of goal-directed behavior: Attitudes, intentions, and perceived behavioral control.. Journal of Experimental Social Psychology.

[B9] Lechner L (1998). Social psychological determinants of health risk behaviors related to cancer and CVD: Applications and elaborations of the ASE model. PhD Thesis.

[B10] Brug J, de Vet E, De Nooijer J, Verplanken B (2006). Predicting fruit consumption: cognitions, intention, and habits. Journal of Nutrition Education and Behavior.

[B11] Kremers SP, Visscher TL, Seidell JC, van Mechelen W, Brug J (2005). Cognitive determinants of energy balance-related behaviours: measurement issues. Sports Medicine.

[B12] Brug J, van Lenthe F (2005). Environmental determinants and interventions for physical activity, diet and smoking: A review.

[B13] Brug J, Lechner L, de Vries H (1995). Psychosocial determinants of fruit and vegetable consumption. Appetite.

[B14] Kok G, de Vries H, Mudde AN, Strecher VJ (1991). Planned health education and the role of self-efficacy: Dutch research. Health Educ Res.

[B15] Cullen KW, Baranowski T, Owens E, Marsh T, Rittenberry L, de Moor C (2003). Availability, accessibility, and preferences for fruit, 100% fruit juice, and vegetables influence children's dietary behavior. Health Educ Behav.

[B16] Kratt P, Reynolds K, Shewchuk R (2000). The role of availability as a moderator of family fruit and vegetable consumption. Health Educ Behav.

[B17] Lien N, Lytle LA, Komro KA (2002). Applying theory of planned behavior to fruit and vegetable consumption of young adolescents.. American Journal of Health Promotion.

[B18] Neumark-Sztainer D, Wall M, Perry C, Story M (2003). Correlates of fruit and vegetable intake among adolescents: Findings from Project EAT. Preventive Medicine.

[B19] Reynolds KD, Hinton AW, Shewchuk RM, Hickey CA (1999). Social cognitive model of fruit and vegetable consumption in elementary school children. Journal of Nutrition Education.

[B20] Hankins M, French D, Horne R (2000). Statistical guidelines for studies of the theory of reasoned action and the theory of planned behaviour. Psychology and Health.

[B21] Yngve A, Wolf A, Poortvliet E, Elmadfa I, Brug J, Ehrenblad B, Franchini B, Haraldsdóttir J, Krølner R, Maes L, Pérez-Rodrigo C, Sjöström M, Thórsdóttir I, Klepp KI (2005). Fruit and vegetable intake in a sample of 11-year-old children in 9 European countries: The Pro Children cross-sectional survey. Annals of Nutrition & Metabolism.

[B22] Sandvik C, Bourdeaudhuij ID, Due P, Brug J, Wind M, Bere E, Pérez-Rodrigo C, Wolf A, Elmadfa I, Thórsdóttir I, Almeida MDV, Yngve A, Klepp KI (2005). Personal, social and environmental factors regarding fruit and vegetable intake among schoolchildren in nine European countries. Annals of Nutrition & Metabolism.

[B23] De Bourdeaudhuij I, Klepp KI, Due P, Pérez-Rodrigo C, de Almeida MDV, Wind M, Krølner R, Sandvik C, Brug J (2005). Reliability and validity of a questionnaire to measure personal, social and environmental correlates of fruit and vegetable intake in 10-11-year-old children in five European countries. Public Health Nutrition.

[B24] Haraldsdóttir J, Thórsdóttir I, de Almeida MDV, Maes L, Pérez-Rodrigo C, Elmadfa I, Frost Andersen L (2005). Validity and reproducibility of a precoded questionnaire to assess fruit and vegetable intake in European 11-to 12-year-old schoolchildren. Annals of Nutrition & Metabolism.

[B25] Kline RB, Kenny DA (2005). Principles and practice of structural equation modeling. Methodology in the Social Sciences.

[B26] Jöreskog KG, Sörbom D (2006). LISREL 8.8.

[B27] Byrne BM (2001). Structural equation modeling with AMOS: Basic concepts, applications and programming.. Multivariate applications book series.

[B28] Loehlin JC (1998). Latent variable models: an introduction to factor, path, and structural analysis.

[B29] Browne MW, Cudeck R, Bollen KA, Long JS (1993). Alternative ways of assessing model fit. Testing structural equation models.

[B30] Foddy W (1993). Constructing questions for interviews and questionnaires: Theory and practice in social research.

[B31] Martens MK, van Assema P, Brug J (2005). Why do adolescents eat what they eat? Personal and social environmental predictors of fruit, snack and breakfast consumption among 12-14-year-old Dutch students. Public Health Nutrition.

[B32] Wind M, De Bourdeaudhuij I, te Velde S, Sandvik C, Due P, Klepp KI, Brug J (2006). Correlates of fruit and vegetable consumption among 11-year-old Belgian-Flemish and Dutch schoolchildren. Journal of Nutrition Education and Behavior.

[B33] Bere E, Klepp KI (2004). Correlates of fruit and vegetable intake among Norwegian schoolchildren: parental and self-reports.. Public Health Nutrition.

[B34] Baranowski T, Cullen KW, Baranowski J (1999). Psychosocial correlates of dietary intake: Advancing dietary intervention. Annual Review of Nutrition.

[B35] Ajzen I (1988). Attitudes, personality and behavior. Mapping social psychology.

[B36] Sutton S (1998). Predicting and explaining intentions and behavior: How well are we doing?. Journal of Applied Social Psychology.

[B37] Reinaerts E, De Nooijer J, Candel M, De Vries N (2007). Explaining school children's fruit and vegetable consumption: The contributions of availability, accessibility, exposure, parental consumption and habit in addition to psychosocial factors. Appetite.

[B38] Blanchette L, Brug J (2005). Determinants of fruit and vegetable consumption among 6-12-year-old children and effective interventions to increase consumption. Journal of Human Nutrition and Dietetics.

[B39] Cullen KW (2003). How good are the measures we obtain? Reliability and validity of the behavioral assessment in the adolescent population. Presentation at Pre-workshop: Assessment of physical activity and nutrition determinants in adolescents: July 17th 2003; Québec City, Canada..

[B40] Blakemore SJ, Frith U (2005). The learning brain: lessons for education.

[B41] Leganger A, Kraft P (2003). Control constructs: Do they mediate the relation between educational attainment and health behaviour?. Journal of Health Psychology.

[B42] Domel SB, Thompson WO, Davis HC, Baranowski T, Leonard SB, Baranowski J (1996). Psychosocial predictors of fruit and vegetable consumption among elementary school children. Health Education Research.

[B43] Bagozzi RP (1992). The self-regulation of attitudes, intentions and behavior. Social Psychology Quarterly.

[B44] Conner M, Sparks P, Conner M, Norman P (2005). Theory of Planned Behaviour and Health Behaviour. Predicting Health Behaviour.

[B45] Armitage CJ, Conner M (2001). Efficacy of the theory of planned behaviour: A meta-analytic review.. British Journal of Social Psychology.

[B46] Fishbein M (2000). The role of theory in HIV prevention. Aids Care.

[B47] Bere E, Brug J, Klepp KI (2007). Why do boys eat less fruit and vegetables than girls?. Public Health Nutr.

